# Risk Perception and Preventive Practice During the COVID-19 Pandemic in the General Population

**DOI:** 10.7759/cureus.36095

**Published:** 2023-03-13

**Authors:** Mahalingam Venkateshan, Priyadarshini Mishra, Satyapriya Mohanty, Asha P Shetty, Gomathi B, Prasanta Kumar Das, Arvind Pandey, Anupama Behera, Dr Debasish Das

**Affiliations:** 1 Nursing, All India Institute of Medical Sciences, Bhubaneswar, Bhubaneswar, IND; 2 Ophthalmology, All India Institute of Medical Sciences, Bhubaneswar, Bhubaneswar, IND; 3 Cardiothoracic Surgery, All India Institute of Medical Sciences, Bhubaneswar, Bhubaneswar, IND; 4 College of Nursing, All India Institute of Medical Sciences, Bhubaneswar, Bhubaneswar, IND; 5 Nursing, Siksha O Anusandhan (Deemed to be University), Bhubaneswar, IND; 6 Anesthesiology and Critical Care, All India Institute of Medical Sciences, Bhubaneswar, Bhubaneswar, IND; 7 Cardiothoracic Surgery, Banaras Hindu University Institute of Medical Sciences, Banaras, IND; 8 Internal Medicine, All India Institute of Medical Sciences, Bhubaneswar, Bhubaneswar, IND; 9 Cardiology, All India Institute of Medical Sciences, Bhubaneswar, Bhubaneswar, IND

**Keywords:** coronavirus, general population, preventive practice, risk perception, covid-19

## Abstract

Background

People’s perceptions of the COVID-19 pandemic and its associated risk are very essential to prevent the spread of the infection. The awareness among individuals may contribute to preventing COVID-19 infections. Coronavirus disease is a serious public health issue. However, preventive practices toward COVID-19 are relatively unknown. The present study aims to survey the risk perception and preventive practice during the COVID-19 pandemic among the general population in Odisha.

Method

A cross-sectional online survey among 395 participants was conducted by adopting the convenience sampling technique. The tools used for the survey consist of three divisions: collection of sociodemographic data, assessment of risk perception toward COVID-19, and assessment of preventive practices during COVID-19 through an online survey method.

Results

The majority (83.29%) of the participants strongly agreed that social distancing is necessary to control the transmission of COVID-19, 65.82% strongly agreed that lockdown is relevant to control COVID-19 spread, 49.62% strongly agreed that wearing a mask protects from the infection, and 40.25% strongly agreed that they will be able to connect with healthcare professionals if they are infected with COVID-19 infection. The finding revealed that the highest number of participants are always practicing all the preventive measures such as maintaining hand hygiene (77.21%), wearing a mask (68.10%), avoiding shaking hands (87.59%), willingness to seek medical help (90.37%), avoiding going to the market or meeting friends (80.75%), discussing preventive measures related to COVID-19 with their family members (76.45%), and eating only homemade food (87.34%).

Conclusion

This study found that an average number of study participants who had the highest level of practice on preventive measures are those who had higher perceived risk among the general population. Expanding the knowledge regarding the infection and its ill effect on health through the proper channel can bring a drastic change in the attitude of the general public. As many people depend on television and social media for acquiring information about COVID-19, any information that reaches the public should be accurate and based on evidence. To avoid miscommunication and the further spread of COVID-19, health education and awareness have to be implemented to increase self-efficacy and risk identification among the general public, which eventually increases the practice of preventive measures.

## Introduction

The coronavirus disease outbreak started in Wuhan, China, in December 2019 and quickly spread to other countries in the world [1]. It was declared a public health emergency by the World Health Organization (WHO) on January 30, 2020, and a pandemic on March 11, 2020 [2].

As per weekly epidemiological data on December 1, 2020, globally the cases of COVID-19 have remained at approximately 4 million new cases, while new deaths have continued to increase to more than 69,000. This brings the cumulative numbers to more than 61.8 million reported cases and 1.4 million deaths globally since the start of the pandemic [2].

As per the Odisha state health department, Bhubaneswar recorded 363 new COVID-19 cases and 3 deaths on December 30, 2020, while 328 patients recovered. Total COVID-19 cases in the state have gone up by 3,26,596. While 3,21,647 cases have been recorded till December 2020, currently, there are 3,057 cases. The total number in the state went up to 1,839 [3].

In Kerala on January 30, 2020, the first case of COVID-19 was detected which rose to three cases by February 3. Interestingly, all three cases were students returning from Wuhan. A nationwide lockdown was imposed from March 2020 to May 2020. Unlocking of the lockdown started on June 1, 2020, in three phases [3,4].

Lack of vaccines and definite antiviral drugs spread the virus in exponential ways for which the use of face masks, hand hygiene, travel restrictions, and social distancing were implemented to curtail the forward transmission of the virus [5-13].

The protection motivation theory states that an individual’s compliance with preventive measures depends on their level of risk perception toward the current health threat. The protection motivation theory stands on four pillars: perceived severity of the threatening event, probability of occurrence, the efficacy of recommended preventive behavior, and perceived self-efficacy [14,15]. Thus, empirical data on how the general population perceives the risks of COVID-19 are essential to devise proper risk communication strategies [5].

The main strategy for the prevention and control of COVID-19 constitutes three factors: wearing a face mask, frequent washing of hands, and maintaining social distancing. Risk perception of people during a pandemic contributes to increasing public participation. Understanding public behavior is critical for the government to develop other effective communication strategies and ensure high compliance with protective behaviors [5].

The perception of risks of COVID-19 and the preventive practices toward it constitute the two most important parameters during the COVID-19 outbreak in the community. Hand washing, maintaining adequate physical distancing, and wearing a face mask are the cornerstones behind the prevention of COVID-19 spread across the community. Risk perception only translates into preventive practice which in turn curtails the community spread of COVID-19 disease. In the present study, we tried to find out the risk perception and preventive practice toward COVID-19, which was the sole determinant of the community spread of the COVID-19 pandemic.

A study was conducted in China, Ethiopia, and the United States, which established risk identification and preventive measures during the COVID-19 pandemic. In India, COVID-19 pneumonia rose to a significant number toward the end of 2020. It was found that the only way to handle this is by creating awareness and removing the social taboos regarding COVID-19 among the public by improving risk perception and preventive practice toward COVID-19 and breaking the chain of infection. Therefore, in this study, we selected the risk perception concerning COVID-19 and preventive practice based on the guidelines issued by WHO for the prevention and control of COVID-19 among the general population.

## Materials and methods

We conducted a cross-sectional study using a self-structured questionnaire developed on Google Forms. Social media channels including e-mail were used to gather a good number of participants. Participants were invited to willingly participate in the survey. To avoid duplication of responses from the same respondent, the survey tool was instructed to accept only one response from the same electronic device at any server. This study was carried out for a one-month period starting from December 1 to December 31, 2021.

Data collection and sampling were conducted online, which gave equal chances to people who have e-mail and social media addresses. The target population in this study included the general public residing in Odisha. A convenience sampling technique was used in this study, and the total number of participants was 395. This study was conducted after the approval of ethical clearance from the Internal Ethical Committee of All India Institute of Medical Sciences (AIIMS), Bhubaneswar. We have included the participants who were willing to participate in the study, and who were able to access the online survey through e-mail and social media platforms. Hence, as this was an online survey method, it gave opportunities to those who have a personal computer or mobile phone with internet facilities. The study excluded those who were unwilling to participate in the study, those who did not have internet facilities and who were not on social media platforms or e-mail addresses, and those who were physically and mentally unfit. As it is an online study, it had its own boundaries and limitation. The validity of the self-structured tool was established by the opinion of the panel of experts. The internal consistency method was used to assess the reliability. The participants were informed about the purpose of the study and gave written consent before the survey. Participation in this study was completely voluntary. Confidentiality of the study was maintained throughout the study.

Survey instrument

A structured questionnaire was developed which contained three divisions: sociodemographic data, risk perception, and preventive practice. The questionnaire was prepared in English and translated into the local language “Odia”.

Sociodemographic Variable

This variable included age, gender, marital status, family type, residence, educational qualification, source of information, and health professional in the family.

Risk Perception

The researcher constructed the questionnaires after reviewing the relevant guidelines from WHO and the Centers for Disease Control and Prevention on risk and preventive measures for COVID-19. A tool was developed to assess the risk perception during the COVID-19 pandemic among the general public using a 5-point Likert scale: strongly agree (5), agree (4), neutral (3), strongly disagree (2), and disagree (1). The participants rated their risk perception of acquiring the COVID-19 infection accordingly. The tool comprised 12 questionnaires, among which six assessed the individual perception of the risk of getting infected by COVID-19 and the rest were related to the community contribution to reducing the spread of COVID-19.

Preventive Practice

The preventive practice was defined by three pillars: hand washing, wearing masks, and physical distancing. The preventive methods during COVID-19 were measured using 12 questions, which were purely based on individuals’ preventive practices to contain the spread and prevent themselves from acquiring COVID-19 infection. These questions were answered on a 5-point Likert scale: always (5), occasionally (4), sometimes (3), rarely (2), and not at all (1). The respondents rated how often they were following the preventive measures.

Data processing and statistical analysis

The data collected were exported to an Excel sheet and checked for its completeness. The Excel data were exported SPSS software (IBM Corp., Armonk, NY) for analysis. Descriptive analysis was applied to calculate frequency and percentage. We used the chi-square test to establish the relationship and significance between variables, i.e., selected demographic variables with the selected risk perception questionnaire and the same set of demographic variables with the selected preventive practice was associated in this study. A p-value of <0.05 was considered statistically significant.

## Results

Sociodemographic data

A total of 395 participants were included in the present study and willingly completed the online survey questionnaire. Of all participants, 70.12% were female and 29.87% were male. The geometric mean age of study participants was 24.6 years. Of the total respondents, 81.26% were married, 76.96% lived in a nuclear family, 51.13% belonged to rural areas, 63.80% completed primary education, 37.97% obtained information through television, and 56.70% did not include healthcare professionals in their family (Table 1).

**Table 1:** Demographic data of the participants (n=395)

Variables	Category	Frequency	Percentage
Age (years)	15-35	373	93.43
	36-55	20	5.06
	56-75	2	0.50
Gender	Male	118	29.87
	Female	277	70.12
	Transgender	0	0
Marital status	Married	321	81.26
	Unmarried	71	17.97
	Single parent	3	0.75
Family type	Nuclear	304	76.96
	Joint	91	23.03
Residence	Urban	202	51.13
	Rural	118	29.87
	Semi-urban	75	18.98
Educational qualification	Primary education	252	63.80
	Secondary education	43	10.89
	Bachelor’s degree	10	2.53
	Master’s degree	81	20.51
	Doctorate degree	2	0.51
	Others	7	1.77
Source of information for COVID-19	Television	150	37.97
	Newspaper	126	31.89
	Internet	87	22.02
	Healthcare professional	26	6.58
	Friend and family	6	1.51
Any healthcare professionals in the family	Yes	171	43.29
	No	224	56.70

Risk perception during COVID -19 pandemic among the general population

All the participants were questioned about their health, the risk of being infected with COVID-19, or the risk of their family being affected by the COVID-19 infection. Among 395 participants, majority (83.29%) of the participants strongly agreed that social distancing is necessary to control the transmission of COVID-19, more than half (65.82%) of the sample strongly agreed that the lockdown is relevant to control COVID-19 spread, 49.62% of the participants strongly agreed that wearing a mask protects them from the infection, and 40.25% of the participant strongly agreed that they will be able to connect with a healthcare professional if they are infected with COVID-19 infection (Tables 2, 3).

**Table 2:** Demographic variables and risk perception during the COVID-19 pandemic (n =395)

Demographic variables	Strongly agree	Agree	Neutral	Strongly disagree	Disagree	X2	p-value
Are you at risk of getting COVID-19 infection?							
Education							
Primary education	47	54	65	50	36	34.25	0.02
Secondary education	9	7	16	8	3		
Bachelor’s degree	2	0	3	3	2		
Master’s degree	5	9	26	22	19		
Doctorate degree	1	0	0	0	1		
Others	3	3	1	0	0		
Is social distancing required to control local transmission of COVID-19?							
Gender							
Male	92	19	4	0	3	10.63	0.03
Female	237	29	9	2	0		

**Table 3:** Demographic variables and preventive practice during the COVID-19 pandemic

Demographic variables	Always	Occasionally	Sometimes	Rarely	Not at all	X2	p-value
Hand washing and use of sanitizer							
Residence							
Urban	171	22	8	0	1	20.79	0.01
Rural	88	21	8	1	0		
Semi-urban	46	22	6	1	0		
Wearing mask							
Gender							
Male	80	21	8	4	5	10.90	0.02
Female	189	56	28	2	2		
Education							
Primary education	178	45	19	5	5	39.47	0.01
Secondary education	26	14	3	0	0		
Bachelor’s degree	6	2	0	0	2		
Master’s degree	50	16	14	1	0		
Doctorate degree	2	0	0	0	0		
Others	7	0	0	0	0		
Maintain distance from family in the home							
Residence							
Urban	79	57	36	13	17	20.32	0.01
Rural	59	28	18	7	6		
Semi-urban	23	13	16	9	14		
Do you avoid going out to the market or meet friends due to COVID-19							
Gender							
Male	85	15	13	4	1	14.54	0.01
Female	234	26	12	1	4		

Preventive practice during COVID -19 pandemic

The government of India and the state government are communicating different preventive measures to avoid COVID-19 infection and minimize losses attributed to the pandemic. Almost 77.21% “always” washed their hands. Overall, 68.10% of participants were wearing masks, 87.59% avoided shaking hands, 90.37% were willing to seek medical help, 80.75% avoided going to the market or meeting friends, 76.45%, and 87.34% ate only homemade food (Tables 2, 3).

**Figure 1:**
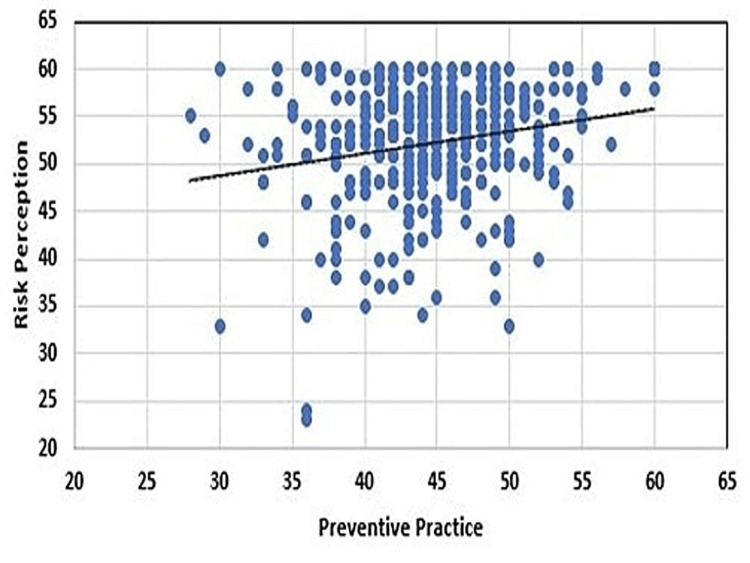
Relationship between preventive practice and risk perception during the COVID-19 pandemic

Tables 2, 3 and Figure 1 depict the association between selected sociodemographic variables and risk perception and preventive practice during the COVID-19 pandemic among 395 participants. Based on the chi-square statistics, sociodemographic characteristics such as education were found to be significantly associated with the risk of getting COVID-19 infection (p = 0.02). There was also a significant association found between gender and physical distance to control local transmission (p = 0.03). Participants' residential area of living such as urban, rural, and semi-urban is significantly associated with the preventive practice of hand washing and use of sanitizer (p = 0.007). Also, educational qualification of participants was significantly associated with wearing masks (p = 0.027). There was a significant association between the area of residence and maintaining social distance (p = 0.009). Also, there was a statistical association between gender and avoiding going out to the market and meeting friends due to COVID-19. The correlation coefficient (r) between risk perception and preventive practice was 0.21. We conclude that there was a weakly positive correlation between the two variables. There is some evidence to suggest that the higher the risk perception, the higher the preventive practice.

## Discussion

The present study was conducted as an effort to understand risk perception and preventive practices among the general population toward COVID-19 in the mid-days of the global pandemic.

The study shows that all the participants had an idea about risk identification and preventive measures for COVID-19. Television, newspapers, telephonic voice record, and various articles were disseminating information on COVID-19 as per the instructions of the government and local authorities. The majority of the participants more often referred to television (37.97%), newspapers (31.89%), internet (22.02%), healthcare professionals (6.58%), and friends and family (1.51%) to get information about the pandemic. In our study, we found a positive association between education and wearing masks (p = 0.03). An Iranian study revealed that persons with low knowledge and education had poor preventive practices against COVID-19 [13].

 Most of the people were aware of the need for social distancing to avoid the transmission of COVID-19 infection (83.29%). In a previous study, among 320 participants, all participants except eight had adequate knowledge about social distance, whereas in this study, 83.29% always maintained social distance during the COVID-19 pandemic, which was a highly appreciable COVID-appropriate behavior.

More than half of the participants agreed that lockdown is necessary for the control of infection. Around 49.62% of participants strongly agreed that the use of wearing masks can protect them from COVID-19 infection. This study on the identification of risk perception in the general public during the COVID-19 pandemic concluded that the highest number of study participants were aware of the risk perception of COVID-19.

The Indian government is enforcing different preventive measures toward minimizing the spread of COVID-19 infection. Our result indicated that 68.10% were using face masks, which is better than Ethiopia, where only 56.5% of the participants wore a face mask to prevent COVID-19. Moreover, avoiding shaking hands (87.59%), avoiding participating in social events such as meeting friends (80.75%), maintaining hand hygiene (77.21%), and eating only home-prepared food (87.34%) as preventive measures are better practiced among our participants than reported in the above study. These better differences in the Indian subset may be due to the enforcement of implementation of preventive practices toward COVID-19 by the Indian government [13].

Similar studies were conducted in China, Ethiopia, and the United States that established risk identification and preventive measures among their citizens. All these studies were conducted when there was a surge in seeking medical help and when cases were rooting up, whereas in India, the morbidity and mortality rate rose to a significant number during the end of 2020. Hence the current study is significant to identify the risk perception and preventive practices during COVID-19 among the general population.

Limitations

The present study has some limitations including randomization through the online system, being cross-sectional in nature, not focusing on healthcare professionals, and having social desirability bias. The participants may overestimate or underestimate the responses in a way that they believe is socially acceptable rather than reporting actual or genuine answers. Convenience sampling was also a limitation. We excluded people who were unwilling to participate in the study, those who did not have internet facilities and who were not on social media platforms or e-mail addresses, and those who were physically and mentally unfit, which was also a major limitation of the study.

## Conclusions

This study shows that the majority of the participants are effectively practicing one or more of the preventive measures to avoid COVID-19 infection, with almost a higher number of participants had been always wearing masks and avoided gathering at 77.21% and 80.75% respectively. Our findings also indicated that the source of information was mainly television followed by newspapers, which may not be available in remote and rural areas. Thus, such residential areas may have less compliance with preventive measures due to a lack of information and knowledge on risk perception. Our study also suggested that social distancing and lockdown as relevant measures to control the spread of infection as the highest number of participants have strongly agreed that these two measures will be helpful in controlling the transmission of infection. There was also a positive significance associated with education and wearing masks. The focus should be on demographic segments with low adoptions, such as younger individuals and those with low education. We conclude that there is a positive correlation between the two variables. There is some evidence to suggest that the higher the risk perception, the higher the preventive practice among the general population. Therefore, effective risk communication and awareness should be implemented to ensure the maintenance of appropriate practice during the COVID-19 pandemic.
